# Coupled molecular and cantilever dynamics model for frequency-modulated atomic force microscopy

**DOI:** 10.3762/bjnano.7.63

**Published:** 2016-05-17

**Authors:** Michael Klocke, Dietrich E Wolf

**Affiliations:** 1 Department of Physics, University of Duisburg-Essen and CeNIDE, D-47048 Duisburg, Germany

**Keywords:** atomic force microscopy, frequency-modulated atomic force microscopy (FM-AFM), energy dissipation

## Abstract

A molecular dynamics model is presented, which adds harmonic potentials to the atomic interactions to mimic the elastic properties of an AFM cantilever. It gives new insight into the correlation between the experimentally monitored frequency shift and cantilever damping due to the interaction between tip atoms and scanned surface. Applying the model to ionic crystals with rock salt structure two damping mechanisms are investigated, which occur separately or simultaneously depending on the tip position. These mechanisms are adhesion hysteresis on the one hand and lateral excitations of the cantilever on the other. We find that the short range Lennard-Jones part of the atomic interaction alone is sufficient for changing the predominant mechanism. When the long range ionic interaction is switched off, the two damping mechanisms occur with a completely different pattern, which is explained by the energy landscape for the apex atom of the tip. In this case the adhesion hysteresis is always associated with a distinct lateral displacement of the tip. It is shown how this may lead to a systematic shift between the periodic patterns obtained from the frequency and from the damping signal, respectively.

## Introduction

The physical background of the dissipation signal in frequency-modulated atomic force microscopy (FM-AFM) was unclear for a long time, and different effects had been discussed, before it was shown that the main contribution comes from adhesion hysteresis [1–3]. However, agreement between theoretical predictions and experimental dissipation rates has remained a challenge [4] in many cases. The detailed reaction path for a given system can be quite complicated, involving different types of hysteresis [5] and additional effects such as lateral tip displacements [6–7].

Computer simulations have been indispensable to disentangle concurrent complex processes that determine the imaging data, i.e., cantilever damping and frequency shift. Roughly, two types of simulations can be distinguished. First, there are simulations of the dynamics of the whole measurement setup [8–9]. They are crucial for understanding experimental parameters and procedures. These virtual AFMs provide a link between a known surface–tip interaction and the resulting images [10–13]. A simplified version of this modeling approach are phenomenological point-mass models of the tip. Their purpose is to clarify basic effects, such as the alternating presence or absence of adhesion hysteresis, if the dissipated energy is not replenished fast enough [14], or the effects of lateral tip displacements on the frequency shift [15] and on the cantilever damping [7]. In all these models, adhesion hysteresis can only be incorporated a priori, e.g., by using different forces on approach and retraction of the tip [16–17].

The second type of simulation models focuses on the atomic dissipation mechanisms of the interaction between tip and sample. Small regions of tip and sample, where the interaction takes place, are simulated with atomic resolution. Some of these models use ab initio molecular dynamics or DFT methods [2,18–19], which represent the material properties most reliably. Their computational demand is very high, though, so that the force between tip and sample surface is usually calculated for a quasistatic tip. Molecular dynamics (MD) simulations [1] with material specific interatomic potentials are computationally less demanding. They can take the finite frequency of the approach and retraction of the tip into account [20–21] and have also been applied to torsional cantilever oscillations, where the tip moves parallel to the sample surface [6]. In these simulations the tip motion is represented in a parametric way, e.g., by prescribing a sinusoidal trajectory normal or parallel to the surface.

Recently situations attracted interest, where “the back-action of the tip–sample force on the cantilever can no longer be ignored” [22]. This is for instance the case, when adhesion hysteresis, lateral and normal cantilever oscillations are coupled with each other, which is the focus of the present paper. Then models such as the one in [22] are needed that are placed in between the above two extremes. To this end, we include the dynamics of the tip in an atomistic simulation. Instead of being guided along a predefined curve, the tip atoms move in a harmonic potential representing the elastic restoring force of the bent cantilever. This has several advantages. The frequency shift and the cantilever damping are simulated directly, and their coupling can be studied. Moreover, the dynamic response of the tip due to its interaction with the substrate allows for the excitation of lateral degrees of freedom, which were suppressed, if the tip trajectory was fixed. They can give rise to a significant contribution to the dissipation signal.

These results can already be obtained for the first cycle of the cantilever oscillation. Focussing on the first cycle allows for a microcanonical approach that is complementary to the common MD simulations, which couple the system to a thermostat. A microcanonical MD simulation has advantages in our case: First, one can avoid the numerical integration of a force hysteresis, so that one gets numerically more accurate values for the dissipated energy. Second, in order to distinguish the two damping mechanisms in a system coupled to a thermostat, one would have to check, if the force hysteresis integrals of the three cartesian components add up to zero (corresponding to energy transfer into lateral degrees of freedom) or not (corresponding to adhesion hysteresis). This is more elaborate and likely more error-prone than monitoring the energies directly.

### Simulation method

**Figure 1:**
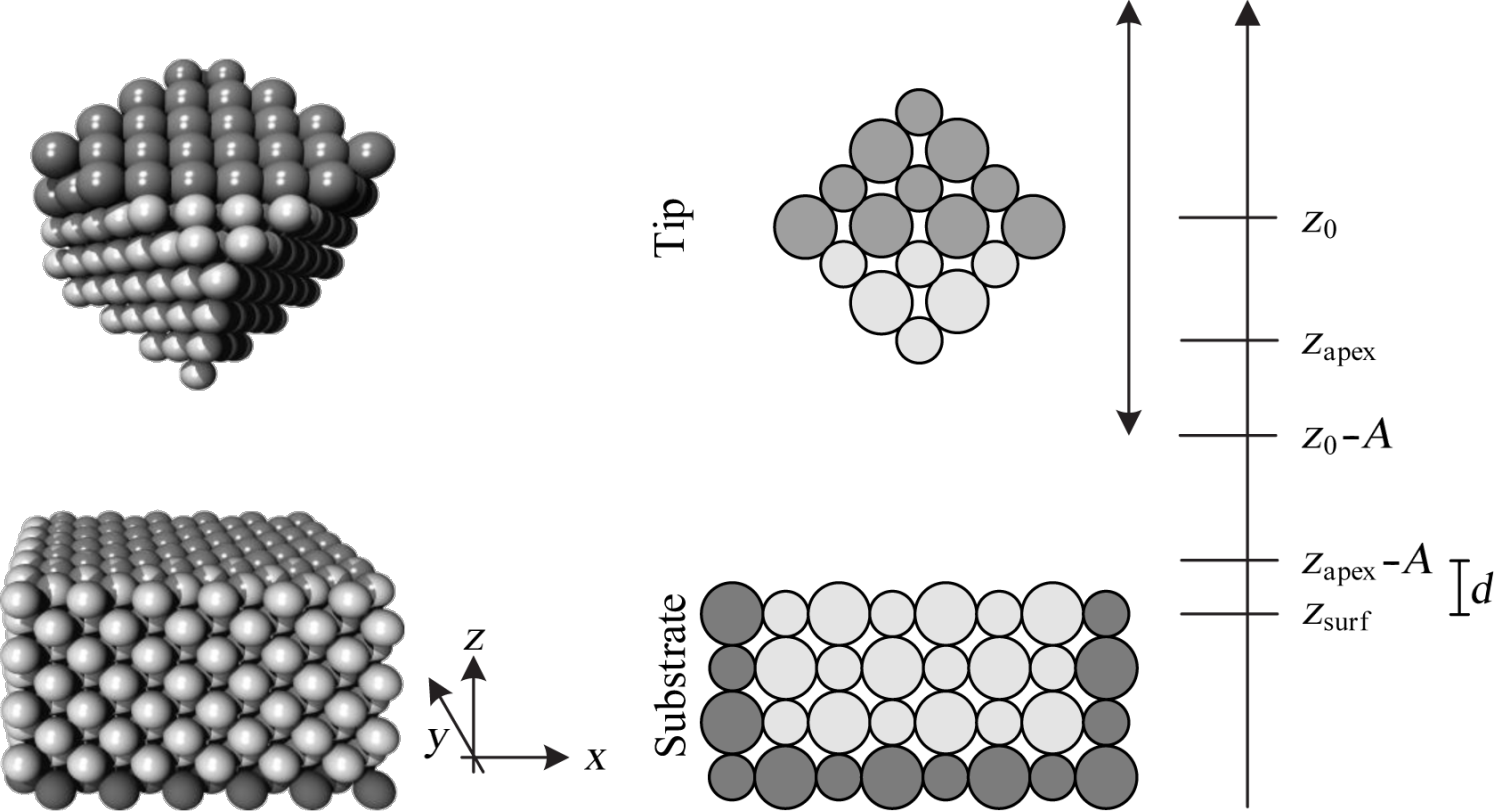
Three-dimensional plot (left) and schematic representation (right) of simulation setup. The tip is represented by a cube, the substrate by a rectangular solid.

It is not feasible to include the full extent of an AFM cantilever nor that of a substrate within an atomistic simulation. As in previous studies [1,6,20–22] the simulation must be restricted to a small volume around the crucial region of interaction between the tip and the substrate. This is sketched in Figure 1. The substrate is reduced to a simple cuboid, and the tip to a cube. Compared to the crystallographic orientation of the substrate, the tip cube is rotated such that one of its diagonal axes is parallel to the *z*-axis. The crystals considered in this paper have face-centered cubic (fcc) or NaCl structure. The *z*-axis corresponds to the [100]-direction in the substrate and the [111]-direction in the tip. There is exactly one apex atom closest to the substrate.

Atoms are treated as point masses with potentials defined between pairs of atoms. They move according to Newton’s equations of motion, which are solved numerically by the velocity Verlet algorithm [23]. The atoms are initially placed according to the stable crystal structure (see below for details). Their movement reflects the collective motion of the tip as a whole, as well as the fluctuations induced by the tip–substrate interaction. This is achieved by several harmonic potentials corresponding to the different modes of the cantilever, and by effective masses of part of the simulated atoms, as described in the next paragraphs.

The bending mode of the cantilever (in the absence of the substrate) can be regarded as a harmonic oscillation. Higher harmonics can usually be neglected as long as they are not excited on purpose [24–26]. Therefore it suffices to let the *z*-component of the center of mass, *z_s_*, of the simulated tip move in a harmonic potential, 
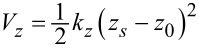
. The equilibrium position of the center of mass is denoted by *z*_0_, and *k_z_* is the stiffness of the bending mode of the cantilever. This potential is applied to each atom of the tip.

As the simulated tip mass *M* is only a small portion of the real cantilever, the experimental values for *k_z_* would lead to an enormously enhanced frequency of the tip oscillation 
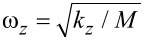
. To obtain a correct frequency of the cantilever, we augment the total mass of the tip *M* by making some atoms “heavier”. However, any impact on the interaction between tip and substrate should be avoided, so that the mass of atoms that are close to the substrate must not be augmented. Therefore the atoms of the tip are divided in two groups, the heavy “head atoms” (dark shade in Figure 1) and the others with normal mass. The mass of the head atoms is augmented by a factor that follows from *k_z_*, ω*_z_*, the normal atomic masses, and the number of head atoms.

The lateral modes can be modeled in a similar fashion. Let *x_s_* and *y_s_* denote the *x*- and *y*-component of the center of mass of the tip and *x*_0_ and *y*_0_ the associated equilibrium positions. The potential


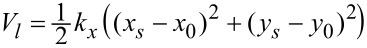


is applied to all atoms of the simulated tip, where *k_x_* denotes the stiffness for the oscillation parallel to the *x*–*y*-plane. Correspondingly, the frequency of the lateral oscillations is given by 
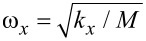
. (*M* is the total mass of the tip including the augmentation factor determined by the calibration of ω*_z_*.) Lateral motion of the tip can be due to torsion of the cantilever axis. The corresponding rotation of the tip around the cantilever axis would imply a slight increase of *z_s_*, correlated to the lateral displacement. However, as the lateral amplitudes are very small compared to the length of the tip, it is justified to neglect this effect.

Due to the rotational invariance of the lateral potential *V_l_*, a restoring torque is missing, when the tip is twisted around the *z*-axis. Therefore, a third harmonic potential is needed that drags all atoms back into their original position relative to the center of mass. In contrast to the other two potentials, this one is only applied to the head atoms. Otherwise it might affect the actual interaction between tip and substrate. For head atom *i* the potential reads


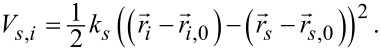


Here, 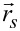 and 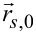 are, respectively, the current center of mass position and the one at the start of the simulation. Likewise, 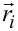 and 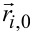 are the current position of atom *i* and the one at the start of the simulation, respectively. Obviously, the potential favors atomic displacements parallel to the one of the center of mass. The stiffness *k_s_* should generally be much larger than *k_z_* and *k_x_*.

Prior to the simulation, the initial configuration is obtained in the following way: First, we set up a system that consists only of the substrate atoms. For the Lennard-Jones crystal, these were placed on regular fcc lattice sites with (100)-surfaces. Then all atoms were allowed to relax according to the finite size of the substrate. Only the *z*-coordinates of the atoms in the bottom layer were fixed. For the (slightly smaller) ionic crystals a relaxation with free boundaries would lead to stronger surface curvature than for the Lennard-Jones crystal. Therefore, a different procedure turned out to be more favorable. For the ionic crystals the substrate ions were placed on a regular NaCl lattice with a lattice constant characteristic for the finite crystal size. Then the positions of all surface atoms apart from those facing the tip were fixed. Note that there is no thermostated layer between the fixed boundary atoms and the ones obeying Newton’s law. In this respect, our simulation differs from the common approach [20].

Next, we set up a system that consists only of the tip atoms. The procedure is the same as used for the substrate with two differences: The tip is rotated as described at the beginning of the section and there are no fixed boundaries. Finally, we assemble the two separate systems by placing the uppermost tip atoms at certain positions relative to the bottom layer of the substrate, while relaxing all emerging stresses. These positions are determined such that the lower apex atom of the tip has the desired lateral position and distance *A* + *d* from the substrate surface *z*_surf_ (Figure 1). We set the equilibrium positions of the harmonic potentials *V_z_* and *V_l_* to the position of the center of mass, and choose the initial velocity of all tip atoms such that the tip would oscillate with amplitude *A*, if the interaction between tip and substrate was switched off. The closest approach to the surface without any interaction between tip and substrate is the nominal distance *d*. If the interaction is switched on, the closest approach will be different.

In this paper we present results for two kinds of atomic interactions. First the Lennard-Jones potential serves as a reference case. Then, a Coulomb interaction is added in order to simulate KBr, which has been studied extensively in experiments [24,27–28].

### Lennard-Jones crystals

If one uses the Lennard-Jones potential,

[1]
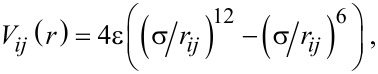


for simulating dynamic force microscopy, the tip in Figure 1 in general does not remain intact, but may loose its apex atom, if it comes too close to the substrate. This can be shown simply by comparing the adsorption energy (the energy, the system gains when an atom is attached to the surface) and the dissociation energy of the apex atom (the energy necessary to detach it from the tip). If the dissociation energy is overcompensated by the adsorption energy, it is more favorable for the apex atom to remain on the substrate. To calculate these energies, we place a single atom onto the surface of the substrate and perform an equilibration run by applying the conjugate gradient algorithm. The difference between the total energy of the relaxed system with and without the atom is the adsorption energy. For a substrate with a size of 6 × 6 × 4 unit cells, we get an adsorption energy of *E_a_* = −5.89ε. Similarly, the dissociation energy *E_d_* = 4.35ε is obtained as difference between the total energy of an isolated tip with and without the apex atom. This shows that the system lowers its energy, if the apex atom detaches from the tip and stays on the surface of the substrate. This is not the case for the potential of KBr described below.

**Figure 2:**
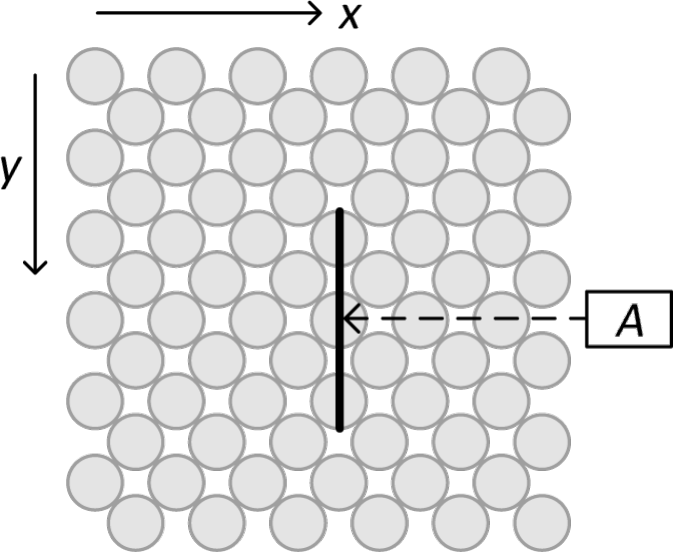
Top view of the substrate surface for the Lennard-Jones case. Distance dependence is probed at position A, whereas constant height scans are performed along the sketched line.

If Lennard-Jones potentials are used exclusively, one way to stabilize the tip is to assume a larger ε parameter for the tip atoms than for the substrate atoms, ε*_t_* *>* ε*_s_*. For the interaction between tip and substrate atoms we use the Lorentz–Bertheloth mixing rule: 
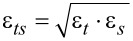
. Stability requires that 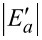 = 5.89ε*_ts_* *<* 4.35ε*_t_* = 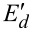, or ε*_t_* *>* 1.84ε*_s_*. To be on the safe side we use ε*_t_* = 2ε*_s_*. All basic parameters for the simulation with Lennard-Jones potentials are shown in reduced units in Table 1. A schematic top view of the substrate surface for this simulation is given in Figure 2.

**Table 1:** Parameter set used for Lennard-Jones case, given in reduced units.

parameter	value

σ	1
*m*	1
ε*_t_*	2
ε*_s_*	1
*k_x_*	2.9·10^−5^
*k_z_*	4.6·10^−7^
*k_s_*	10^4^
ω*_x_*	3.4·10^−4^
ω*_z_*	4.3·10^−5^
*A*	5
substrate size	6 × 6 × 4 unit cells (576 atoms)
tip size	4 × 4 × 4 unit cells (256 atoms)

### Ionic crystals: Lennard-Jones plus Coulomb

As a simple model for ionic crystals, we add the Coulomb interaction to the Lennard-Jones potential as in [15]:

[2]
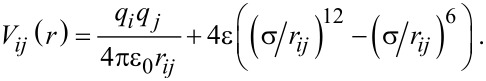


**Figure 3:**
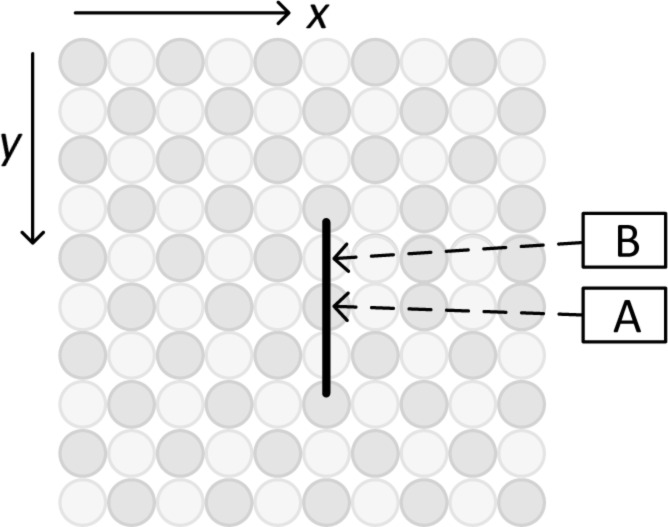
Top view of the substrate surface for the ionic crystal. Distance dependence is probed at position A with oppositely charged apex and projection atom and at position B with same charges. Constant height scans are performed along the sketched line.

Here, it is not necessary to use different Lennard-Jones parameters for the tip and the substrate, because the dissociation energy for the apex atom is not overcompensated by the adsorption energy. The parameters are chosen such that the lattice constant of a KBr crystal is reproduced, and that the error of the cohesive energy is less than 5%. They are given in Table 2, this time in SI units in order to facilitate comparison with previous works and experimental findings. Figure 3 provides the corresponding schematic top view of the substrate surface.

**Table 2:** Parameter set used for the ionic case.

parameter	value

σ	0.361 nm
ε	0.011 eV
*m*_K_	39 u
*m*_Br_	79 u
*k_x_*	64 N/m
*k_z_*	4 N/m
*k_s_*	4.8·10^4^ N/m
*f_x_*	400 MHz
*f_z_*	1600 MHz
*A*	1.8 nm
substrate size	5 × 5 × 3 unit cells (600 atoms)
tip size	3 × 3 × 3 unit cells (216 atoms)

Equation 2 is a very simple potential for ionic crystals. Often, more sophisticated Buckingham-type pair potentials [21] are used, which have an exponential short range repulsive part instead of the one with the 12th power in Equation 2. Also, the parameters are different for K–K, Br–Br and K–Br interactions. A further refinement takes the electronic polarization into account via a shell model replacing the rigid ions [29]. It must be kept in mind, though, that the atomic interaction is only one among several important factors that influence FM-AFM simulations. For example the shape of the tip can change the dissipation rate by as much as 100% [21]. For our present purpose, to investigate qualitatively, what the effect of concurring adhesion hysteresis and lateral excitations is, the simple potential is justified.

### Determination of dissipation rate Δ*E* and frequency shift Δ*f*

Experimentally the dissipation rate is determined as the amount of energy necessary to maintain a fixed amplitude of the cantilever oscillation. Correspondingly we monitor the energy of the normal oscillation of the center of mass of the tip, *z_s_*, during the half cycle, when it approaches and retracts from the substrate surface. At *t* = 0 the tip starts at the equilibrium position *z_s_* = *z*_0_ and returns to the same *z*-coordinate at *t* = *t*_end_. The damping of the normal cantilever oscillation per cycle is then given by

[3]
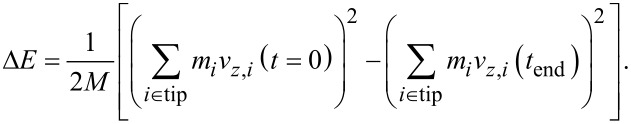


As the calculations are microcanonical, the energy of the whole system is conserved. Δ*E* must be balanced by an increase of energy in another part. As far as dissipation is concerned, this part would be the inner degrees of freedom of the crystal (that is, substrate and tip are heating up). It is also possible that the energy of another macroscopic degree of freedom is increased, e.g., by a lateral excitation of the tip. For this reason, Δ*E* is called “damping” rather than “dissipation”.

The frequency shift is evaluated as

[4]
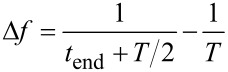


with the unperturbed oscillation period *T* = 1/*f_z_*.

## Results and Discussion

### Lennard-Jones crystal

**Figure 4:**
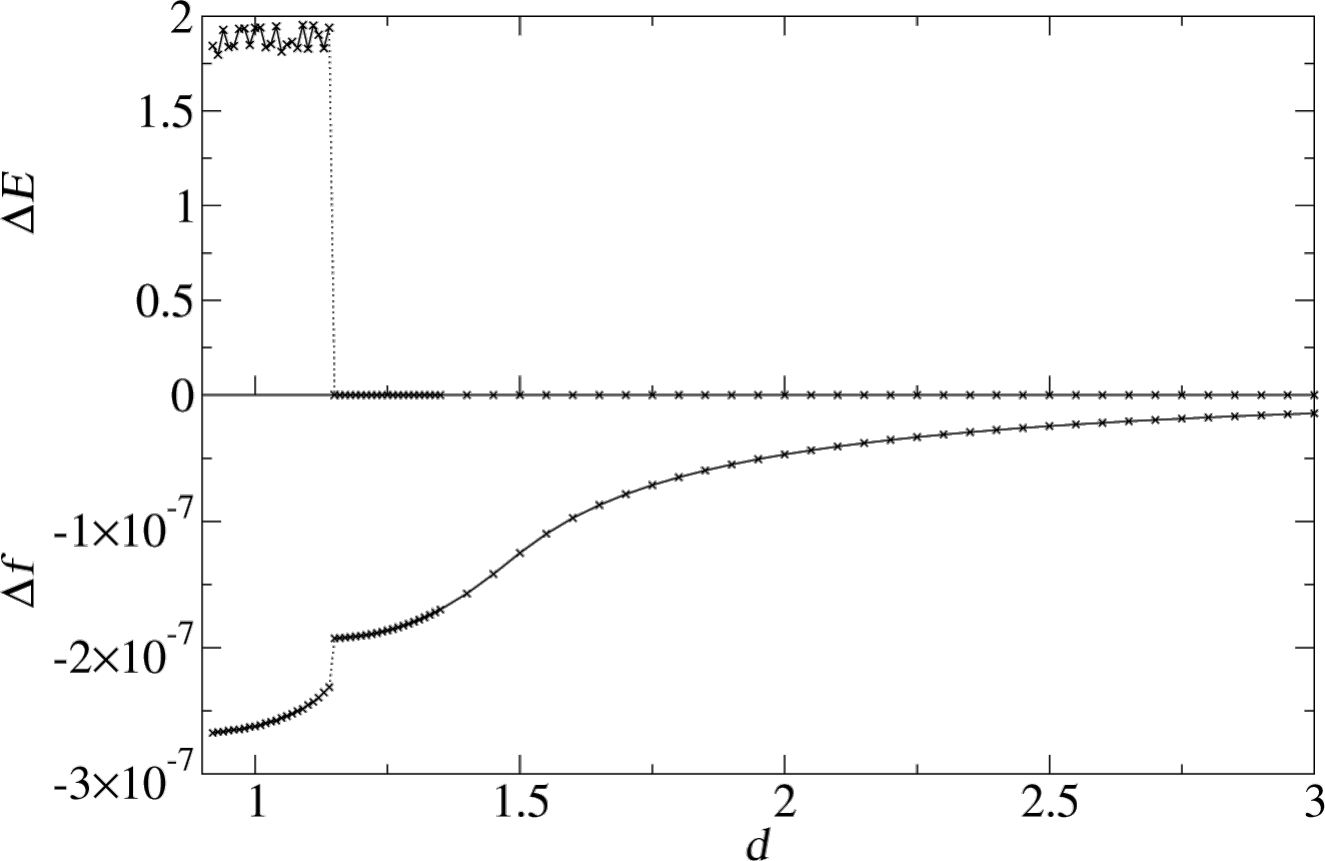
Energy dissipation and frequency shift as a function of the nominal distance. Each point is calculated by a single simulation run with shifted *z*_0_. Lateral tip position is set to A of Figure 2.

**Figure 5:**
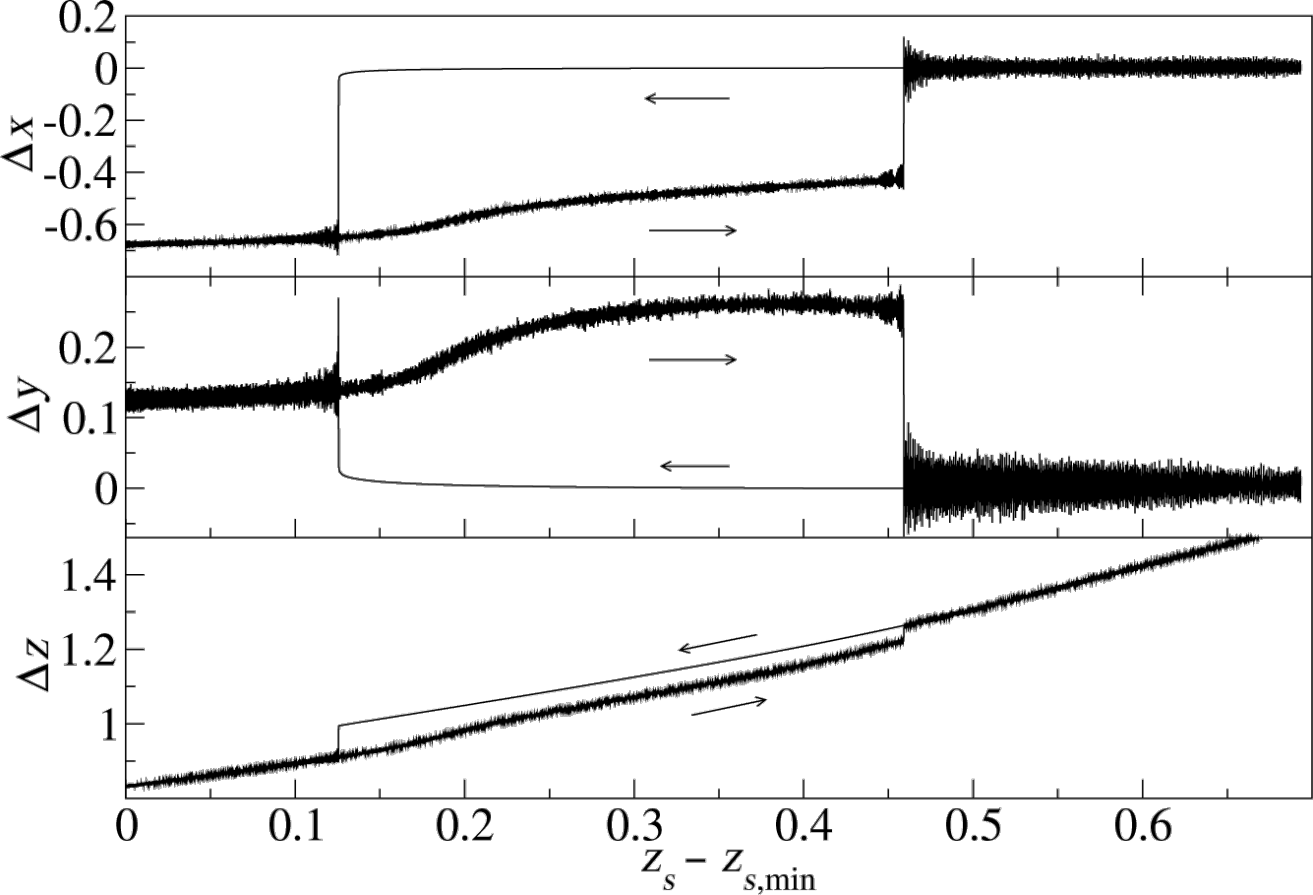
Trajectory of the apex atom at tip position A (Figure 2). Δ*x* and Δ*y* are its *x*- and *y*-coordinates relative to their values at time *t* = 0. The *z*-coordinate Δ*z* is given relative to *z*_surf_.

Figure 4 shows the dissipation and the frequency shift as a function of the nominal distance *d* between tip and surface for a Lennard-Jones crystal. The apex atom is directly above an atom of the substrate (position A in Figure 2). Energy dissipation sets in abruptly as soon as the nominal distance drops below *d <* 1.2 and is basically constant for smaller distances. This is a typical sign for adhesion hysteresis: The sudden configurational change happens only if the tip comes close enough to the surface. Once it happened, the system will go through the hysteresis loop upon retraction of the tip (as shown in Figure 5). The hysteresis is independent of the smooth, elastic deformation of the configuration, when the tip approaches the surface further before being retracted. Therefore the dissipation is independent of the closest approach. The onset of dissipation can be seen so clearly here, because substrate and tip start with zero temperature at *t* = 0. At finite temperatures the onset of dissipation becomes smeared out due to thermal fluctuations.

One strength of the present model is that it allows for a direct determination of the frequency shift. As expected, a decrease of the nominal distance reduces the frequency (the shift is negative), because of the attraction between tip and surface. It has been predicted [16,18] that the beginning of the dissipation also leads to a change in frequency shift. This is confirmed by the lower plot of Figure 4: The frequency shift shows a discontinuity at the nominal distance where dissipation sets in. The sharp jump will be smeared out for higher temperatures. Comparing the size of the discontinuity to the continuous part of the frequency shift, it can be estimated that adhesion hysteresis is responsible for approximately 20% of the frequency shift. This is rather large, and although the precise ratio clearly depends on the potential used, it is important to realize that the frequency shift is generally not independent of the dissipation signal.

This coupling between the dissipation and the topology signal has further consequences, which are observable experimentally. In order to explain them, a closer inspection of the energy landscape of the apex atom is needed. Figure 5 shows the coordinates of the apex atom for a tip oscillating with a nominal distance of *d* = 1.05 above point A in Figure 2. The actual minimal distance is smaller than *d* due to the attractive forces between tip and substrate. The trajectory shows a hysteresis loop with significant displacements in all three dimensions.

As the tip starts with zero temperature, the apex coordinates first do not fluctuate, when the tip approaches the substrate. However, as soon as the apex atom jumps into its new stable position, the released energy is dissipated, heating the substrate and the tip. From then on the apex coordinates fluctuate. On retraction, the apex atom jumps back, when its original position becomes more favorable again and the energy barrier between the two minima becomes small enough. The energy difference between the two minima is dissipated again, enhancing the apex fluctuations further.

**Figure 6:**
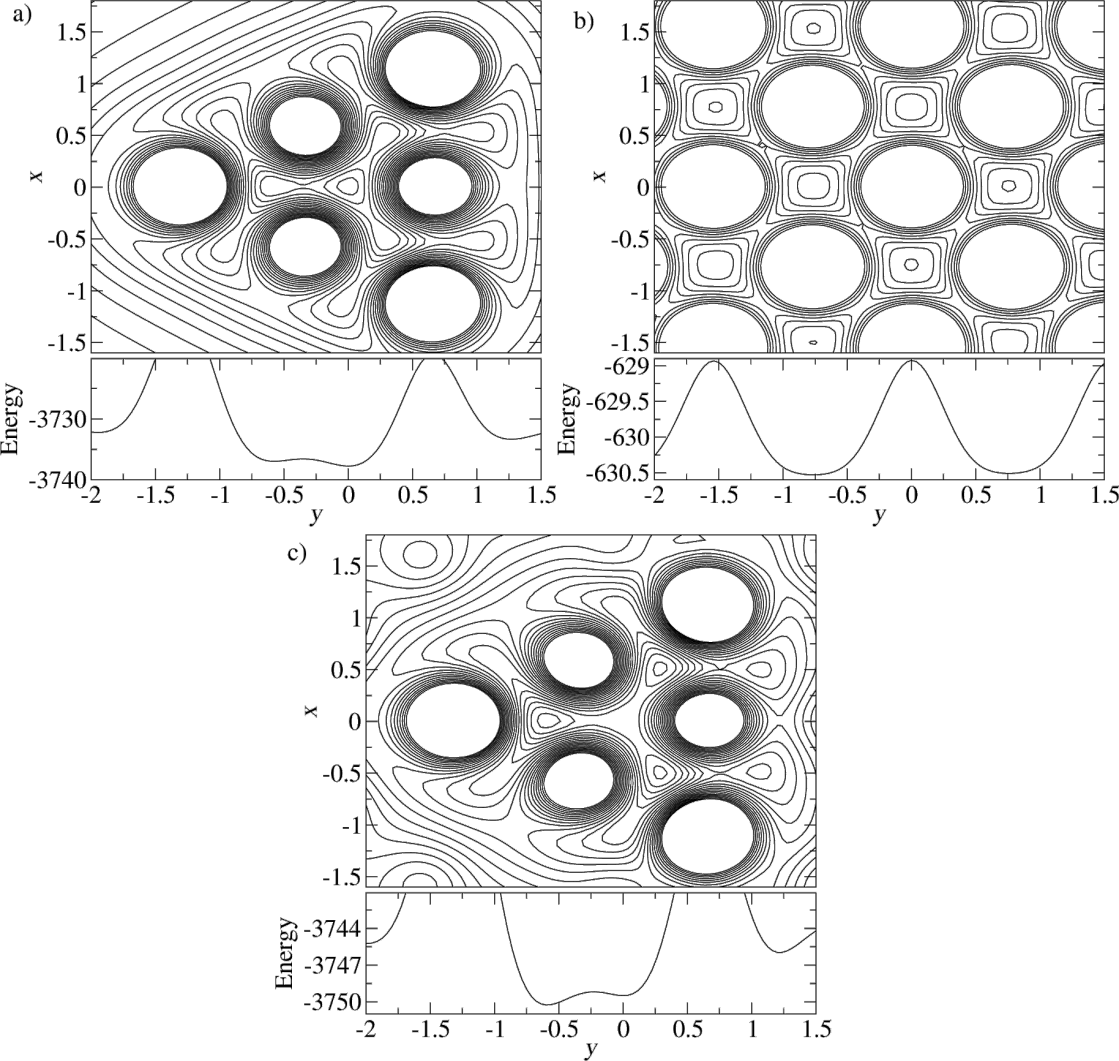
Potential energy surface a) in the (111)-plane of the apex atom, when the tip is far away from the substrate; b) for an adatom in a plane above the substrate; c) in the (111)-plane of the apex atom when the tip is near the substrate (*d* = 1.05). The graphs under the contour plots show the corresponding cross-section of the potential energy surface for *x* = 0.

In order to analyze the configurational change associated with the adhesion hysteresis, we probe the potential landscape by relocating the apex atom parallel to the *x*–*y*-plane (Figure 1), while the rest of the configuration remains fixed (in particular the distance between tip and substrate). In the first panel of Figure 6, tip and substrate are far apart. The diagram shows a contour plot of the energy landscape in the *x*–*y*-plane with origin at position A (Figure 2). High energy values are cut in such a way that the strongly repulsive regions appear as large white areas (these belong to the next neighbors of the apex atom). Beneath the contour plot, the energy variation along the *y*-axis for *x* = 0 is shown. As expected, the total minimum can be found at *y* = 0. Analogous plots are shown in the second panel of Figure 6, but calculated in an *x*–*y*-plane very close to the surface of the substrate. As we have placed the apex atom directly above an atom of the substrate, one finds a strong repulsion at *x* = 0, *y* = 0.

Not so close, but still at a small distance to the substrate, one obtains the last panel of Figure 6. The energy landscape changes distinctly as both substrate and tip contributions are important. One can roughly view this energy landscape as the sum of the two previous ones. With this picture, the instability leading to the hysteresis gets more clear: While the original apex position at the center becomes unstable due to the repulsion by the substrate atom, the other minima are less affected, as they are rather near a regular lattice site for an adatom on the surface. This explains why the minimum of the energy landscape for the apex atom at *x* = 0, *y* ≈ −0.6 is now lower than the one at *x* = 0, *y* = 0 (see the graph under panel c of Figure 6). However, this is not the minimum the apex atom jumps to. It is off the y-axis roughly in (61−1)-direction as concluded from Figure 5.

The coupling between the dissipation and the topography signals combined with the lateral displacement in the adhesion hysteresis explains an effect that has been a puzzle for some time [30]: A systematic discrepancy between the positions of frequency and dissipation maxima, even for a system as simple as the Lennard-Jones crystal considered here. We performed simulations of a scan, where the tip probes many *y*-positions within an interval of three lattice constants (as shown in Figure 2). The nominal distance *d* was kept fixed during such a scan (similar to the “constant height”-mode of an AFM).

**Figure 7:**
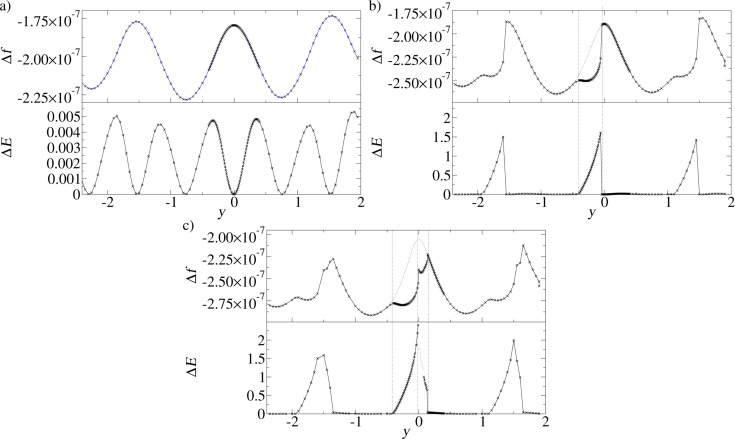
Simulated constant-height scans along the line shown in Figure 2 for a Lennard-Jones system (position A at *y* = 0). The nominal distances are a) *d* = 1.30, b) *d* = 1.22, c) *d* = 1.12. Frequency shift and damping of the cantilever are shown. For b) and c) adhesion hysteresis occurs near position A. The dissipation signal is correlated with a frequency drop. For c) there is an interval to the right of position A, where the atomic configuration of the tip changes irreversibly (grey), i.e., Δ*E* is the energy dissipated in the first cycle only.

In Figure 7 the frequency shift and the dissipation (with atomic resolution) are compared for three such scans with different nominal distances *d*. For the largest distance, *d* = 1.30, the hysteresis is not present. Hence the damping is only minute (note the different Δ*E*-scale compared to the panels for the other two *d*-values). It is due to an energy transfer into the lateral degree of freedom. A signature of this effect is that it is nearly equally strong on both sides of the points of strongest attraction between tip and substrate, so that the scan has two dissipation maxima per lattice constant, but only one frequency minimum. In the next section, an example will be discussed, where this effect is stronger.

Figure 7b shows the scan lines for *d* = 1.22. The closer approach of the tip implies a stronger attraction, hence a lower frequency. Moreover, adhesion hysteresis is present in a fraction of the unit cell. It leads to more than 100 times stronger dissipation maxima than in the first panel. Whereas outside the *y*-intervals with significant dissipation the qualitative *y*-dependence of the frequency is unchanged, it is strongly reduced, where adhesion hysteresis occurs. This means that the system jumps into a configuration with enhanced attraction. The asymmetric shape of the dissipation curve can be understood with the help of the potential energy surfaces shown in Figure 6a,b. Consider the tip at position *y* = 0, which is directly above a substrate atom A. The attraction between tip and substrate is minimal, because the apex atom is far from an fcc binding site to the substrate. As the tip moves in *y*-direction, the fcc binding site to the right of atom A moves towards the center of the triangle of the potential energy surface in Figure 6a, stabilizing the apex atom. When the tip has passed the fcc binding site, the secondary potential minimum left of the apex position benefits from it. This is when the bistability of the apex atom emerges, first with a very small hysteresis. However, the hysteresis increases, as the tip moves towards the substrate atom to the right of A, and the lateral jump of the apex atom into the substrate-induced minimum becomes larger and larger. This explains why the maximal dissipation is reached almost at the point of weakest attraction between tip and substrate. The asymmetry of the dissipation curve is a direct consequence of the mismatch between the triangular potential landscape of the tip and the square one of the substrate.

In Figure 7c the tip is even closer to the substrate, with a nominal distance of *d* = 1.12. In the *y*-interval, where dissipation occurred already in the previous panel, the curves for dissipation and frequency remain qualitatively the same. However, dissipation extends now also further to the right. The adhesion hysteresis here takes place on the other side of the point of weakest attraction between tip and substrate, and has the opposite asymmetry than before. The explanation is, that now the secondary minima in Figure 6a, which are off the *y*-axis, are the jump destinations. This adhesion hysteresis again causes a frequency reduction. Due to the inverted symmetry, the position of maximal adhesion hysteresis is no longer close to the frequency maximum. It can be assumed that this effect is rather general, whenever adhesion hysteresis involves lateral displacements in different directions. A systematic shift between dissipation and frequency shift has indeed been observed in experiments [30–31].

### Ionic crystal

In order to link the conceptual findings of the previous section to a real system, an ionic crystal KBr, we include a long-range Coulomb part in the potential (Equation 2) and give all parameters and results in SI units. The system setup is similar to the one of the previous section.

**Figure 8:**
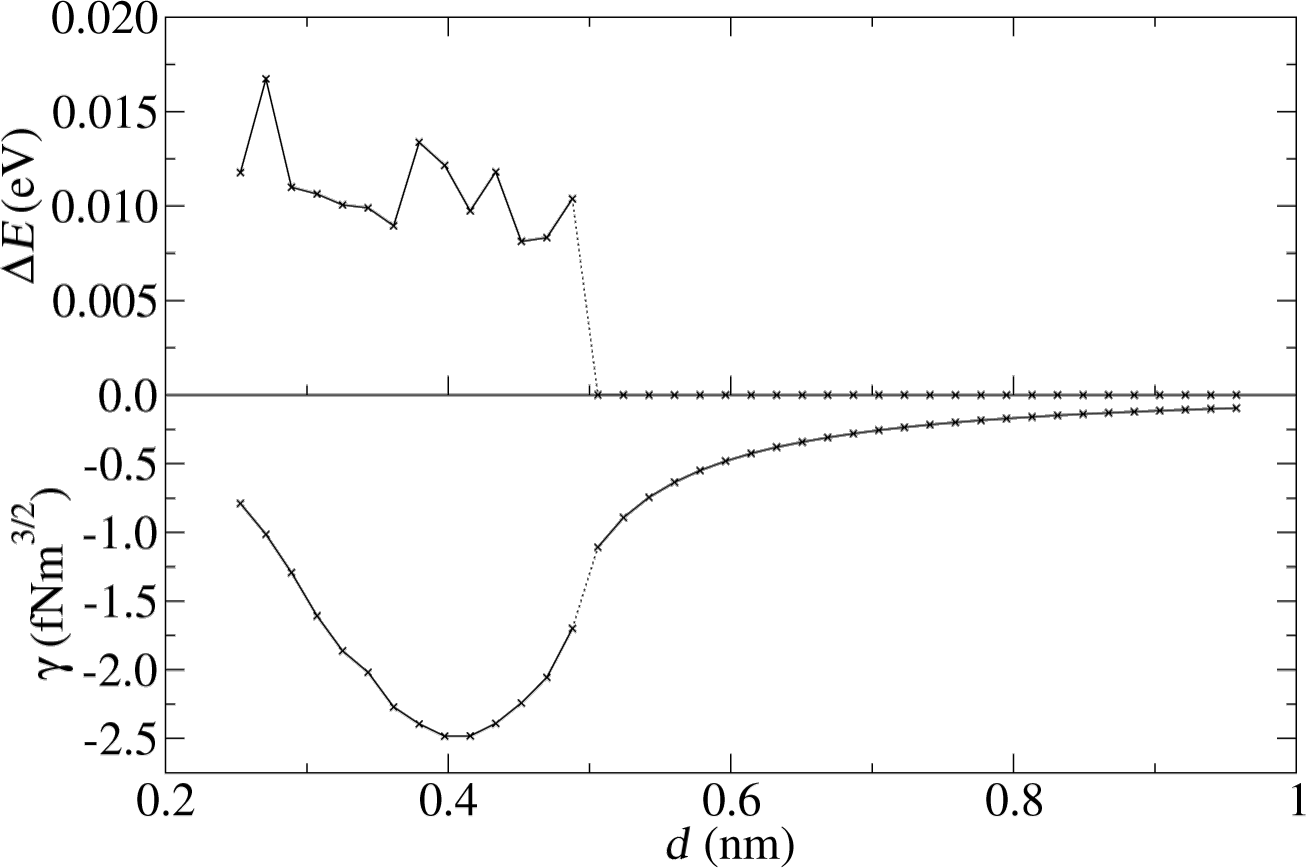
Damping and reduced frequency shift as a function of the nominal distance *d* for position A (apex atom and projection atom are oppositely charged).

Figure 3 shows the surface of the ionic crystal. First we let the tip descend over position A, where the apex atom and the projection atom directly underneath are oppositely charged. The results, Figure 8, are clear indication of adhesion hysteresis: energy dissipation sets in abruptly as soon as the tip approaches the substrate closer than (nominally) 5.4 Å. At that point, also the reduced frequency shift γ = (Δ*f*/*f_z_*)*k_z_A^3/2^* changes abruptly. (The reduced frequency shift is commonly used instead of Δ*f* in order to minimize the dependence on system parameters such as *f_z_* or *k_z_*. This is helpful in the simulation context, because molecular dynamics simulations are only feasible with exaggerated frequencies *f_z_* [32]). As in Figure 4, the dissipated energy Δ*E* fluctuates around a roughly constant value, if the tip approaches the substrate close enough that the adhesion hysteresis occurs. The reason is the same: The simulation starts with zero temperature. After the sudden configurational change during the tip approach the temperature increases, so that the position, at which the original configuration is resumed upon retraction of the tip, has thermal fluctuations.

The results for frequency shift and dissipation are similar to those discussed for the simple Lennard-Jones system in Figure 4, although the configurational changes are completely different: Here it is the projection atom that undergoes a significant displacement. The hysteresis loop for the apex atom is much smaller. Another difference is the lack of lateral displacement. This can be understood by the local potential, which the apex atom sees in the rock salt structure. The (111) atomic plane (parallel to the (100) substrate surface) next to the apex atom consists of only three atoms of opposite charge, in contrast to the fcc structure (Figure 6a), where it consists of six atoms. Besides the potential minimum, in which the apex atom of a KBr tip sits, there are no secondary minima, which could become favorable due to the substrate influence. Hence the asymmetry of the tip with respect to the substrate has little dynamical effect. This explains, why no lateral displacement can be observed at positions A and B, where the apex atom descends directly above an attractive (A) or repulsive (B) substrate atom.

**Figure 9:**
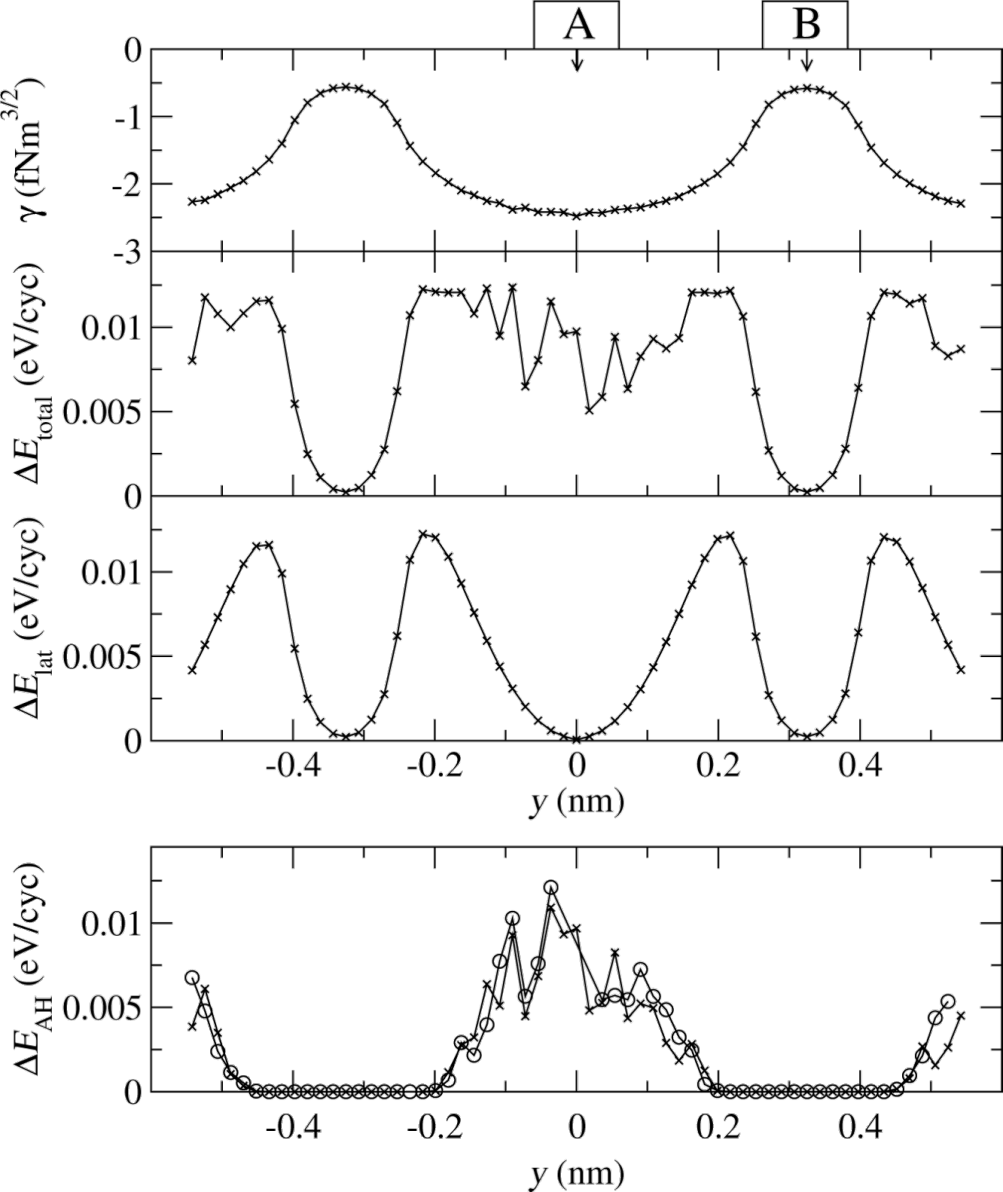
Simulated constant-height scan (*d* = 0.4 nm) for KBr along the line shown in Figure 3. The first panel shows the frequency shift, the second shows the total damping rate Δ*E*_total_, which is composed of the damping rate due to lateral oscillations (Δ*E*_lat_ shown in the third panel) and due to adhesion hysteresis (Δ*E*_AH_ shown in the fourth panel). The fourth panel also shows the dissipation rate, if the excitation of lateral oscillations is suppressed (open circles).

Along the line connecting the positions A and B of Figure 3 both the hysteresis as well as the lateral excitation occur, separately or simultaneously to varying degrees. This is the conclusion drawn from Figure 9. The first panel shows the positional dependence of the reduced frequency shift obtained with the parameters of Table 2 and *d* = 0.4. The second panel shows the damping of the cantilever for the same simulations. In order to discriminate between the two damping mechanisms, the third panel displays the energy ending up in the lateral degrees of freedom *x_s_* and *y_s_* of the center of mass of the tip. It nearly vanishes, if the apex atom is above a substrate atom, but has pronounced maxima in between. Subtracting the third from the second panel, one obtains the energy dissipated due to adhesion hysteresis, panel four. It is the dominant mechanism around tip position A, but vanishes near tip position B. The regions, where both mechanisms are present, overlap. There, both mechanisms contribute to the damping on the same order of magnitude.

This raises the question, whether or not the two damping mechanisms enhance or inhibit each other. In order to answer this question we repeated the simulation with the only difference that the lateral stiffness *k_x_* was enhanced by a factor of 10^4^, thereby essentially suppressing the excitation of lateral oscillations. Then the cantilever damping is solely determined by the adhesion hysteresis. It is also shown in the fourth panel of Figure 9. Within the numerical errors it agrees with the difference curve obtained from the second and third panel. This proves that adhesion hysteresis is not affected significantly by the excitation of lateral oscillations, at least in the present case of KBr.

**Figure 10:**
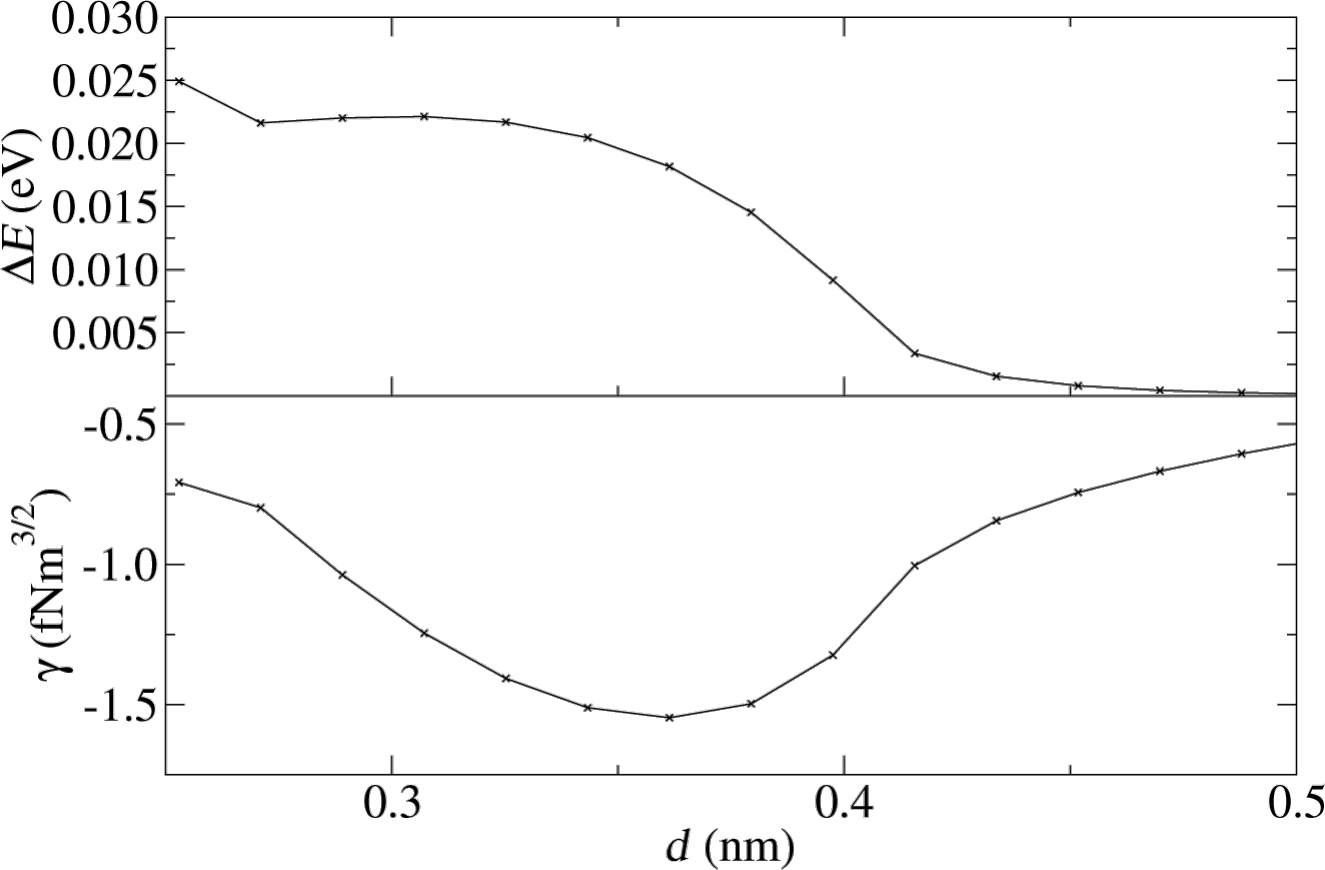
Damping and reduced frequency shift as a function of the nominal distance *d* for position *y* = 0.243 nm, where the damping is exclusively due to lateral excitations.

Now the tip is placed at position *y* = 0.243, where according to Figure 9 exclusively lateral excitations are responsible for the cantilever damping. Figure 10 shows a completely different *d*-dependence of the dissipation behavior. It is not step-like as in Figure 8. The excitation of the lateral oscillation of the tip continuously becomes stronger when the nominal distance between tip and substrate decreases. Previously, we pointed out the conceptual difference between the damping of the tip motion and an actual dissipation (see [7] for a detailed discussion). If we evaluate the dissipation directly (the energy of the internal degrees of freedom without the motion of the tip as a whole), we find that it actually vanishes. This shows that the energy is transferred into the macroscopic lateral modes rather than being distributed randomly among the internal degrees of freedom.

The cantilever damping is determined by the energy landscape of the system and therefore depends on the parameter ε in Equation 2, which quantifies the relative strength of the short-range Lennard-Jones interaction compared to the long-range Coulomb interaction. It will vary for different ionic crystals with rock salt structure.

**Figure 11:**
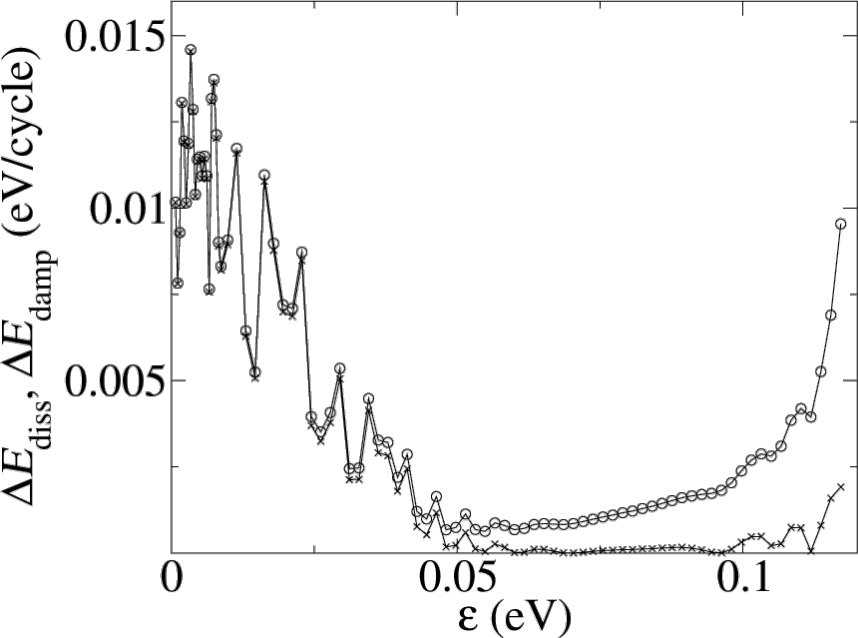
Damping rate (upper curve) and dissipation rate (lower curve) as a function of the strength ε of the short range part of the potential. Below 0.05 eV the cantilever damping is due to adhesion hysteresis. Above, it is mainly due to the transfer of energy from the normal into the lateral oscillation modes.

The ε-dependence of the cantilever damping at point A of Figure 3 for a nominal distance *d* = 0.4 nm is shown in Figure 11. The figure shows also the dissipation, which accounts only for a part of the damping. The difference is the energy transferred into the lateral degrees of freedom. For increasing ε the damping becomes weaker first, nearly vanishes near ε ≈ 0.05 eV, but then increases again. This remarkable re-occurring damping can be traced back to the two different damping mechanisms (adhesion hysteresis or lateral excitation). Up to ε ≈ 0.05 the damping is entirely due to adhesion hysteresis. As already discussed for KBr, lateral modes are not excited in significant amounts. However, the adhesion hysteresis becomes weaker and ultimately ceases to exist: The increased short-range attraction makes it more and more difficult to let the projection atom jump from the substrate towards the approaching tip. The high coordination number wins over the point-like long distance attraction between apex and projection atom.

For larger ε, the damping of the normal cantilever oscillation is dominated by its coupling to the lateral modes. Dissipation of energy to the internal degrees of freedom hardly occurs. How can this finding be reconciled with the argument presented above, that no lateral excitation should be possible at point A? The essence of that argument was that the apex atom of the tip sits in a deep potential well without secondary local minima around, so that its position in the *x*–*y*-plane remains essentially unchanged, when the interaction between tip and substrate is switched on. However, the point contact force between tip and substrate also increases with ε, hence also its lateral components, if there are any. In fact the total potential energy of substrate and tip has a lower symmetry than for the substrate or the tip separately. This leads to a lateral excitation of the tip without adhesion hysteresis.

## Conclusion

Simulating the motion of the cantilever together with the atomistic dynamics led to several new insights relevant for frequency-modulated atomic force microscopy. Specifically, we considered ionic crystals with rock salt structure. The interaction between pairs of ions was modeled by the Coulomb potential plus a short range contribution of the Lennard-Jones type. The AFM tip and the substrate were of the same material. The (100)-surface was probed by a cubic portion of the tip (with (100) faces). The tip was turned such that its diagonal direction was normal to the sample surface.

This class of models shows adhesion hysteresis, when the apex ion of the tip is directly above an oppositely charged ion of the substrate surface. However, the dissipation becomes weaker, if the short range attraction between the atoms is increased, and vanishes above a critical Lennard-Jones parameter ε ≈ 0.05 eV. Then a different mechanism of the cantilever damping takes over: Energy is transferred from the normal cantilever oscillation into the lateral modes without any adhesion hysteresis. This transition from one damping mechanism to another one can only be simulated with a molecular dynamics model that includes the dynamics of the cantilever.

The two damping mechanisms can be distinguished by their dependence on the nominal distance *d* between the tip and the substrate. For the adhesion hysteresis the dissipation sets in abruptly as soon as *d* becomes small enough and remains roughly constant below. At the same time, the frequency of the cantilever oscillation changes discontinuously. If the normal cantilever oscillation is damped, for lateral oscillations get excited, the energy transfer increases smoothly with decreasing *d*, due to the increasing lateral forces, and the normal frequency has no discontinuity. This correlated behavior of the frequency shift and the damping rate can be observed in experiments.

For a KBr (100) surface we could show that there exist different tip positions, where either one of the two damping mechanisms dominates, and those, where both occur simultaneously. Adhesion hysteresis was found at and around the tip position, where the apex atom of the tip faces a substrate ion of opposite charge. These correspond to binding sites of the ionic crystal. Lateral cantilever excitation was found in an annulus around the position where these charges are equal. In between, both mechanisms contribute significantly to the cantilever damping.

Even the simple Lennard-Jones crystal shows adhesion hysteresis. In contrast to KBr, the adhesion hysteresis does not occur close to the binding sites of the substrate, but at the least attractive positions. It is always connected with lateral displacement of the apex atom. A careful analysis of the potential landscape for the apex atom explains this behavior and reveals the reason, why the dissipation signal is sometimes systematically shifted by a fraction of a lattice constant into one direction compared to the frequency signal. Turning the argument around, this observation gives indirect information about the combined energy landscape of tip and substrate, and hence about the tip geometry.

These results show that the combined simulation of atomic and cantilever dynamics is very instructive and leads to a better understanding of frequency modulated atomic force microscopy. In this paper we studied KBr type systems, because such ionic crystals were among the earliest samples where the mechanism of adhesion hysteresis was investigated experimentally [33] and theoretically [1]. However, by now, many more examples are known with distinct adhesion hysteresis, notably also organic molecules with covalent bonds [34]. These systems are chemically more complex, but the principal mechanism of adhesion hysteresis is the same as for a simple KBr surface. Also, lateral displacements of the tip should not only occur in ionic crystals. Therefore a combination of both causes for cantilever damping should be expected for other systems, as well. When interpreting the experimental data one has to be aware of this.
